# Near complete genome sequence of the animal feed probiotic, *Bacillus amyloliquefaciens* H57

**DOI:** 10.1186/s40793-016-0189-z

**Published:** 2016-09-06

**Authors:** Benjamin J. Schofield, Adam Skarshewski, Nancy Lachner, Diane Ouwerkerk, Athol V. Klieve, Peter Dart, Philip Hugenholtz

**Affiliations:** 1 School of Agriculture and Food Sciences, The University of Queensland, St Lucia, QLD Australia; 2 Australian Centre for Ecogenomics, School of Chemistry and Molecular Biosciences, The University of Queensland, St Lucia, QLD Australia; 3 Department of Agriculture and Fisheries, Dutton Park, QLD Australia; 4 Queensland Alliance for Agriculture and Food Innovation, The University of Queensland, St Lucia, QLD Australia

**Keywords:** *Bacillus amyloliquefaciens*, Probiotic, Antimicrobials, Illumina

## Abstract

*Bacillus amyloliquefaciens* H57 is a bacterium isolated from lucerne for its ability to prevent feed spoilage. Further interest developed when ruminants fed with H57-inoculated hay showed increased weight gain and nitrogen retention relative to controls, suggesting a probiotic effect. The near complete genome of H57 is ~3.96 Mb comprising 16 contigs. Within the genome there are 3,836 protein coding genes, an estimated sixteen rRNA genes and 69 tRNA genes. H57 has the potential to synthesise four different lipopeptides and four polyketide compounds, which are known antimicrobials. This antimicrobial capacity may facilitate the observed probiotic effect.

## Introduction

*Bacillus amyloliquefaciens species have* been taxonomically classified as part of the *Bacillus subtilis* group. Members of this group share substantial morphological similarities and near identical (98.1 %–99.8 %) 16S rRNA gene sequences [1]. Other members of the *Bacillus subtilis* group include *B. subtilis*, *B. atrophaeus*, *B. licheniformis*, *B. sonorensis*, *B. tequilensis*, *B. vallismortis*, and the *B. mojavensis* subgroup. The production of bioactive metabolites, the ability to form spores and a lack of pathogenicity make members of the *Bacillus subtilis* group ideal candidates for use as probiotics. Strains of *B. amyloliquefaciens* synthesise non-ribosomal bioactive lipopeptides such as surfactin, fengycin, bacillomycin D and members of the iturin family [2–4]. These lipopeptides have demonstrated activity as antimicrobials and inhibit a wide range of bacterial and fungal pathogens [3, 5].

The strain *B. amyloliquefaciens* H57 (H57 hereafter) was first isolated in the search for a biological control agent to prevent fungal spoilage of hay [6]. Due to its spore forming ability and production of antimicrobial compounds, H57 was revealed as the best candidate of a panel of isolates for commercialisation as a spoilage control agent under the product name HayRite™. Importantly, sheep and cattle fed on HayRite™ treated feed showed an increase in digestibility and nitrogen retention leading to increased live weight gain [6]. This new development into the potential of H57 to act as a probiotic has led to further investigation of this strain.

Here, we present a summary description of the classification and features of H57, along with a sequencing description and annotation summary. The availability of a genome sequence for H57 will facilitate research into the probiotic effects observed in animals treated with this bacterium.

## Organism information

### Classification and features

**Fig. 1:**
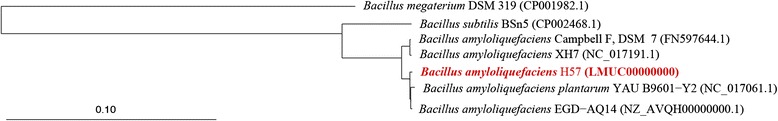
Maximum likelihood tree showing the alignment of H57 with other *Bacillus* genomes. Alignment was performed using HMMER [8] whilst maximum likelihood was inferred using FastTree version 2.7.7 [32]. The inferred tree was visualised using ARB version 6.0.2 [33]. Bar: 0.1 substitutions per nucleotide position

A near-complete 16S rRNA gene was identified in the H57 genome, which by BLAST [7] is most closely related (99 % identical) to other *B. amyloliquefaciens* strains including FZB42 (*B. amyloliquefaciens* subsp. *plantarum*; acc. NR075005.1), HPCAQB14 (acc. KF861603.1) and SB 3200 (acc. GU191911.1). Comparison of the average read coverage of the genome and 16S rRNA gene, suggests that H57 has 13 copies of the rRNA operon. A concatenated alignment of 99 single copy marker genes obtained from publicly available *Bacillus* genomes using HMMER [8] confirmed the classification of strain H57 as a member of the species *B. amyloliquefaciens* (Fig. 1).

**Fig. 2:**
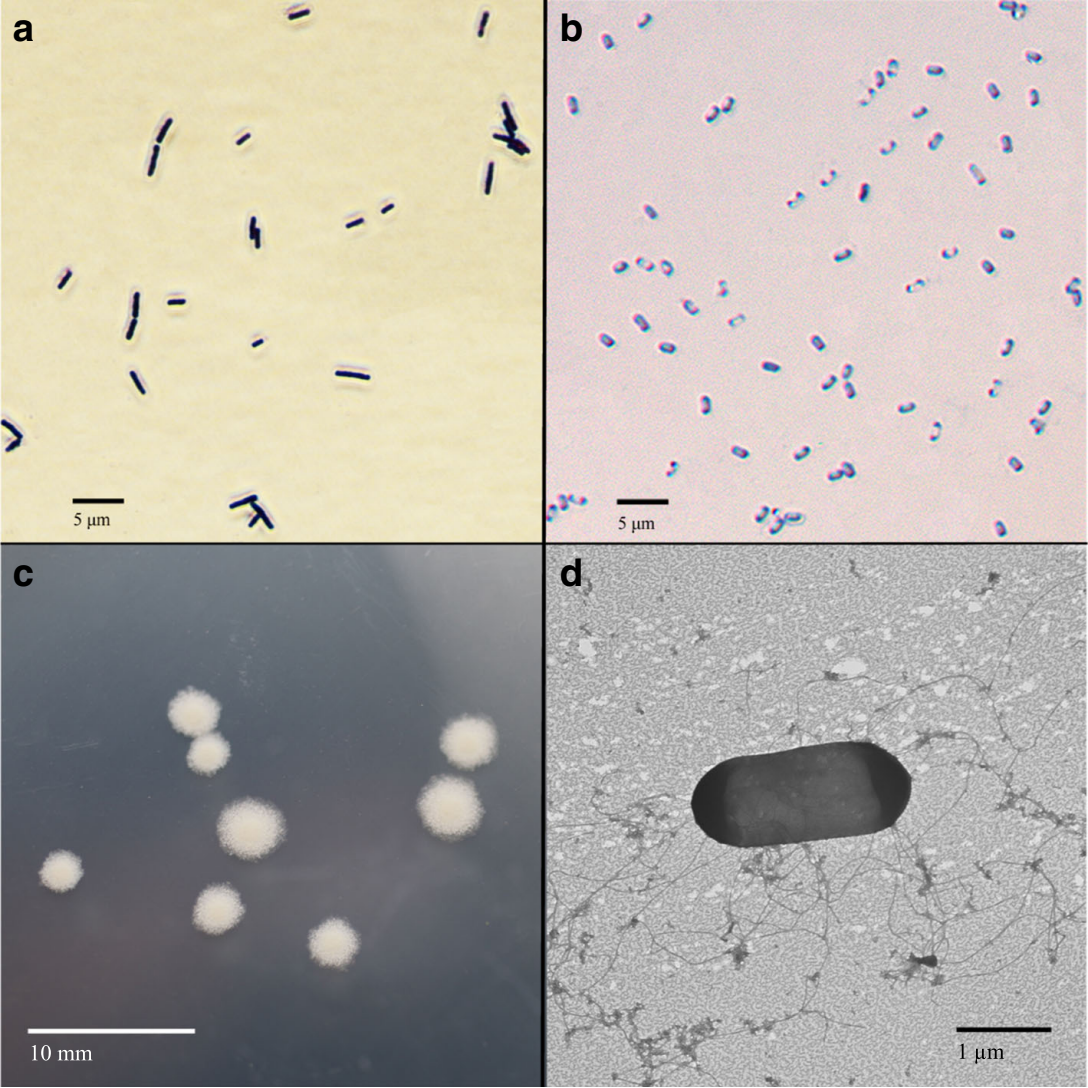
Cellular and colony morphology of *B. amyloliquefaciens* H57. **a** Vegetative H57 cells at 1000x magnification captured with a Nikon DS-Ri1 camera attached to a Nikon Eclipse 80i microscope under phase contrast. **b** H57 spores at 1000x magnification captured with a Leica DFC 500 camera attached to a Leica DM5500B compound microscope with Nomarski differential interference contrast. **c** Pure culture of H57 grown on nutrient agar plate. **d** Electron microscope image of a vegetative H57 cell showing numerous peritrichous flagella, negatively stained with phosphotungstic acid

H57 is a Gram-positive rod shaped bacterium averaging 2.5 μm in length and 1 μm in width (Fig. 2d). It is an aerobic spore forming bacterium that is motile with peritrichous flagella. H57 spores are centrally located and average 1.25 μm in length (Fig. 2b). Optimum growth occurs at a temperature of 29 °C and pH 7.0 (Table 1). The colony morphology of strain H57 is circular convex with undulate margins. When grown on a nutrient agar plate, colonies are an off-white colour as shown in Fig. 2c.

**Table 1:** Classification and general features of *Bacillus amyloliquefaciens* strain H57 [10]

MIGS ID	Property	Term	Evidence code^a^
	Classification	Domain *Bacteria*	TAS [34]
		Phylum *Firmicutes*	TAS [35–37]
		Class *Bacilli*	TAS [38, 39]
		Order *Bacillales*	TAS [40, 41]
		Family *Bacillaceae*	TAS [40, 42]
		Genus *Bacillus*	TAS [40, 43, 44]
		Species *Bacillus amyloliquefaciens*	TAS [45–47]
		Strain: H57	
	Gram stain	Positive	IDA
	Cell shape	Rod/chains	IDA
	Motility	Motile	IDA
	Sporulation	Sporulating	IDA
	Temperature range	Mesophilic	IDA
	Optimum temperature	29 °C	IDA
	pH range; Optimum	5-9; 7	IDA
	Carbon source	Glucose, fructose, mannitol, sucrose, trehalose	IDA
MIGS-6	Habitat	Leaves of *Medicago sativa*	TAS [6]
MIGS-6.3	Salinity	Up to 6 % (w/v)	IDA
MIGS-22	Oxygen requirement	Aerobe	IDA
MIGS-15	Biotic relationship	Symbiotic (beneficial)	TAS [6]
MIGS-14	Pathogenicity	Non-pathogen	NAS
MIGS-4	Geographic location	Gatton, QLD, Australia	IDA
MIGS-5	Sample collection	2001	IDA
MIGS-4.1	Latitude	27° 32' 24'' S	IDA
MIGS-4.2	Longitude	152° 20' 24'' E	IDA
MIGS-4.4	Altitude	89 m	IDA

^a^ Evidence codes - *IDA* Inferred from Direct Assay, *TAS* Traceable Author Statement (i.e., a direct report exists in the literature), *NAS* Non-traceable Author Statement (i.e., not directly observed for the living, isolated sample, but based on a generally accepted property for the species, or anecdotal evidence). These evidence codes are from the Gene Ontology project [48]

## Genome sequencing information

### Genome project history

Strain H57 was selected for sequencing due to its ability to act as a probiotic in agricultural animals. The draft genome was deposited in GenBank under the accession number LMUC00000000. Genome sequencing and assembly was performed at the Australian Centre for Ecogenomics, The University of Queensland. Gene annotation was performed using the AnnotateM script [9]. A summary of the project is shown in Table 2 using MIGS version 2.0 [10] criteria.

**Table 2:** Project information

MIGS ID	Property	Term
MIGS 31	Finishing quality	Draft
MIGS 28	Libraries used	Illumina paired end library (256 bp insert size)
MIGS 29	Sequencing platforms	Illumina MiSeq
MIGS 31.2	Fold coverage	49×
MIGS 30	Assemblers	Spades 3.0.0.
MIGS 32	Gene calling method	PROKKA
	Locus tag	Ga0082361
	Genbank ID	LMUC00000000
	GenBank Date of Release	04/04/2016
	GOLD ID	Ga0082361
	BIOPROJECT	PRJNA300579
MIGS 13	Source material identifier	*Bacillus amyloliquefaciens* H57
	Project relevance	Probiotic, Agriculture

### Growth conditions and genomic DNA preparation

Genomic DNA of H57 was isolated from a freeze-dried product of H57 spores combined with sodium bentonite (1:1). DNA was extracted from the H57 spores using the ‘Repeated Bead-beating and Column Extraction’ method described by Yu and Forster (2005) [11]. In brief, 0.1 g of sporulated product was added to 1 mL of lysis buffer (2.9 % NaCl, 0.6 % Tris, 0.05 M EDTA pH 8.0 and 4 % SDS) in a cryotube containing 0.5 g zirconia beads (BioSpec Products Inc., Bartlesville, USA). The sample was then homogenised in a mini bead beater 16 (BioSpec Products Inc., Bartlesville, USA) for 2 cycles of 3 min. Between cycles the samples were incubated for 15 min at 70 °C, centrifuged (13,200 rpm for 5 min at 4 °C) and supernatant transferred to a fresh tube. Following bead beating further extraction was performed on the supernatant using the QIAGEN QIAmp DNA Mini Kit as per kit instructions (QIAGEN, Doncaster, VIC).

### Genome sequencing and assembly

The genome of H57 was sequenced on an Illumina MiSeq sequencing platform (Illumina, Inc. San Diego, CA). DNA libraries were prepared using the Nextera® XT DNA Library Preparation Kit (Illumina, San Diego, CA) according to the manufacturer’s instructions. An input of 1 ng was used to prepare DNA libraries, which was then cleaned using Agencourt AMPure XP beads (Beckman Coulter, Brea, CA, USA). The purified PCR product was then size selected for amplicons with a size between 300 bp and 800 bp. Illumina paired-end sequencing was performed, producing a total of 1,351,526 reads. Primer and adaptor sequences were removed using Trimmomatic v0.32 [12] resulting in an average read length of 256 bp. Reads were assembled using SPAdes 3.0.0. [13]. The H57 genome was obtained in 16 contigs ranging in size from 701,147 bp to 10,158 bp with a combined length of 3,958,833 bp. Genome completeness and contamination was estimated using CheckM version 1.0.0, indicating that the genome was near complete (99.51 %) with no detectable contamination (0 %) [14].

### Genome annotation

Gene annotation was achieved using a combination of protein databases via AnnotateM Version 6.0 [9]. Open reading frames were initially generated using PROKKA [15]. The resulting protein sequence was then searched against the IMG, Uniref, COG, PFAM and TIGRfam databases [16–20] to identify homologous genes. The software ProteinOrtho [21] was used to identify orthologous genes to other known *B. amyloliquefaciens* strains for further comparison. Genes unique to H57 were compared against the KEGG gene database [22] to identify metabolic functions.

### Genome properties

The draft genome assembly of H57 consists of sixteen contigs totalling 3,958,833 bp and a G + C content of 46.42 %, which is likely a slight underestimate of its genome size due to unresolved collapsed repeats, primarily rRNA operons (Table 3). With a coding region of 3,549,557 bp, this assembly represents a total of 3,945 ORFs. Of those genes, 3,836 encode proteins and the remainder encode sixteen rRNAs (7 × 5S, 7 × 16S and 2 × 23S), 69 tRNAs and 24 other RNA genes (Table 3). Of the annotated genes, the majority were assigned a putative function (80.66 %) with 69.81 % assigned into Clusters of Orthologous Groups, presented in Table 4. Of the 3,945 ORFs in the H57 genome, 3,751 were inferred to be orthologous to other *B. amyloliquefaciens* strains, including strains CC178, DSM7, XH7, TF28, Y2, IT-45, LFB112 and *B. amyloliquefaciens* subsp *plantarum* strains UCMB5113, FZB42, NAU-B3, YAU B9601-Y2, and TrigoCor1448. Of the 194 genes unique to H57, several appear to be involved in the degradation of aromatic compounds, more specifically the breakdown of 4-hydroxyphenylacetic acid.

**Table 3:** Genome statistics

Attribute	Value	% of Total
Genome size (bp)	3,958,833	100.00
DNA coding (bp)	3,549,557	89.66
DNA G + C (bp)	1,837,549	46.42
DNA scaffolds	16	100.00
Total genes	3,945	100.00
Protein coding genes	3,836	97.24
RNA genes	109	2.76
Pseudo genes	0	0.00
Genes with internal clusters	387	9.81
Genes with function prediction	3,182	80.66
Genes assigned to COGs	2,754	69.81
Genes with Pfam domains	3,364	85.27
Genes with signal peptides	191	4.84
Genes with transmembrane helices	1,046	26.51
CRISPR repeats	0	0.00

**Table 4:** Number of genes associated with general COG functional categories

Code	Value	%age^a^	Description
J	136	3.48	Translation, ribosomal structure and biogenesis
A	0	0.00	RNA processing and modification
K	89	2.23	Transcription
L	95	2.43	Replication, recombination and repair
B	1	0.03	Chromatin structure and dynamics
D	22	0.56	Cell cycle control, Cell division, chromosome partitioning
V	17	0.44	Defence mechanisms
T	58	1.48	Signal transduction mechanisms
M	97	2.48	Cell wall/membrane biogenesis
N	40	1.02	Cell motility
U	37	0.95	Intracellular trafficking and secretion
O	64	1.64	Posttranslational modification, protein turnover, chaperones
C	92	2.35	Energy production and conversion
G	109	2.79	Carbohydrate transport and metabolism
E	160	4.09	Amino acid transport and metabolism
F	62	1.59	Nucleotide transport and metabolism
H	93	2.38	Coenzyme transport and metabolism
I	53	1.36	Lipid transport and metabolism
P	93	2.38	Inorganic ion transport and metabolism
Q	30	0.77	Secondary metabolites biosynthesis, transport and catabolism
R	203	5.19	General function prediction only
S	238	6.09	Function unknown
-	2169	55.49	Not in COGs

^a^The total is based on the total number of protein coding genes in the genome

### Insights from the genome sequence

Comparative analysis of the H57 genome indicates that its central metabolism is consistent with other strains of *B. amyloliquefaciens*. The presence of a complete TCA cycle and electron transport chain indicates the potential for aerobic respiration. H57 has *a narGHJI* operon and the transcriptional regulator *fnr*, suggesting that it is also capable of growing anaerobically using nitrate as an electron acceptor [23]. This capability would be required for H57 to grow in anoxic environments.

The genome of H57 also encodes a number of enzymes involved in carbohydrate metabolism. A search against the carbohydrate-active enzyme database [24] reveals that H57 is dominant in glycoside hydrolase families 1, 43 and 13 (Table 5). The GH 1 and GH 43 families comprise enzymes that degrade the various sugar monomers of hemicellulose. This suggests that H57 may contribute to breaking down the less fibrous components of the plant cell wall. The abundance of GH 13 enzymes, which are a family of α-amylases, suggests that H57 also contributes to the breakdown of starch. The presence of these carbohydrate-activated enzymes alludes to the notion that H57 may assist in the digestion of animal feeds by breaking down certain polysaccharides of the plant cell wall.

**Table 5:** Carbohydrate activated enzyme profile of glycoside hydrolases in H57

Family	Known activity	%^a^
GH16	Xyloglucan, keratan-sulfate, endo-1,4-β-galactosidase, endo-1,3- β-glucanase, and others	2.5
GH4	Maltose-6-phosphate glucosidase, α-glucosidase, α-galactosidase, and others	7.5
GH5	Chitosanase, β-mannosidase, cellulase, glucan 1,3-β-glucosidase, and others	2.5
GH13	α-amylase, pullulanase, cyclomaltodextrin glucanotransferase and others	10
GH11	Xylanase	2.5
GH23	Lysozyme type G and peptidoglycan lyase	2.5
GH3	β-glucosidase, xylan 1,4-β-xylosidase, β-N-acetylhexosaminidase, and others	2.5
GH126	Other	2.5
GH18	Chitinase, endo-β-N-acetylglucosaminidase, and others	7.5
GH26	β-mannanase and β-1,3-xylanase	2.5
GH53	Endo-β-1,4-galactanase	2.5
GH51	α-L-arabinofuranosidase and endoglucanase	5
GH1	β-glucosidase, β-galactosidase, β-mannosidase, and others	12.5
GH73	Peptidoglycan hydrolase with endo-β-N-acetylglucosaminidase specificity	5
GH30	Glucosylceramidase, β-1,6-glucanase, β-xylosidase	5
GH32	Endo-inulinase, endo-levanase, exo-inulinase, and others	7.5
GH46	Chitosanase	2.5
GH109	α-N-acetylgalactosaminidase	5
GH43	Arabinases and xylosidases	10
GH68	Levansucrase, β-fructofuranosidase and inulosucrase	2.5
	Total GH hits:	40
	Total ORFs:	3,828
	% GH ORFs:	1.04

^a^Percentage of total GH hits

Consistent with observed anti-fungal activity, the H57 genome encodes a broad range of antimicrobial compounds. These include genes for non-ribosomal synthesis of antimicrobial lipopeptides such as surfactin (*srfABCD*), iturin (*ituABCD*), bacillomycin D (*bmyABC*) and fengycin (*fenABCDE*). Surfactin is capable of inhibiting a wide range of microorganisms due to its ability to insert itself into the cell wall creating ion pores [25]. Bacillomycin D, iturin and fengycin all have demonstrated antifungal properties primarily based on their ability to disrupt the fungal cell wall [26–28]. The genes for the expression of antibiotic polyketides are also present on the H57 genome. These include the operons *mlnABCDEFGHI*, *dfnABCDEFGHIJ* and *baeEDLMNJRS*, which encode macrolactin, difficidin and bacillaene respectively. These compounds inhibit a wide range of microorganisms acting chiefly on preventing protein synthesis [29–31].

## Conclusions

The ~3.96 Mbp genome of *B. amyloliquefaciens* H57 reveals the basis of its antimicrobial nature and potential to survive and reproduce in anoxic animal gastrointestinal tracts. In common with other *B. amyloliquefaciens* strains, H57 encodes a wide range of antimicrobial compounds that explain its effectiveness as a biocontrol agent for fungi and other feed spoilage organisms. The production of these compounds may also contribute to the observed probiotic effect by inhibiting potentially pathogenic organisms creating a healthier microbial ecosystem.

## Abbreviations

GH: Glycoside hydrolase
